# Age, Tumor Characteristics, and Treatment Regimen as Event Predictors in Ewing: A Children's Oncology Group Report

**DOI:** 10.1155/2015/927123

**Published:** 2015-10-05

**Authors:** Neyssa Marina, Linda Granowetter, Holcombe E. Grier, Richard B. Womer, R. Lor Randall, Karen J. Marcus, Elizabeth McIlvaine, Mark Krailo

**Affiliations:** ^1^Department of Pediatrics, Stanford University and Lucile Packard Children's Hospital, 1000 Welch Road, Suite 300, Palo Alto, CA 94304-1812, USA; ^2^Department of Pediatrics, New York University, Langone Medical Center, New York, NY 10016, USA; ^3^Pediatric Hematology-Oncology, Dana Farber & Boston Children's Hospital, 44 Binney Street, Boston, MA 02115, USA; ^4^Division of Oncology, Children's Hospital of Philadelphia, Philadelphia, PA 19104, USA; ^5^Sarcoma Services, Huntsman Cancer Institute and Primary Children's Medical Center Department of Orthopaedics, University of Utah, Salt Lake City, UT 84112, USA; ^6^Department of Radiation Oncology, Boston Children's Hospital/Dana Farber Cancer Institute Brigham and Women's Hospital, Harvard Medical School, Boston, MA 02115, USA; ^7^Department of Preventive Medicine, University of Southern California, Los Angeles, CA 90027, USA; ^8^Children's Oncology Group Statistics, Monrovia, CA 91016, USA

## Abstract

*Purpose*. To associate baseline patient characteristics and relapse across consecutive COG studies. *Methods*. We analyzed risk factors for LESFT patients in three randomized COG trials. We evaluated age at enrollment, primary site, gender, tumor size, and treatment (as randomized). We estimated event-free survival (EFS, Kaplan-Meier) and compared risk across groups (log-rank test). Characteristics were assessed by proportional hazards regression with the characteristic of interest as the only component. Confidence intervals (CI) for RR were derived. Factors related to outcome at level 0.05 were included in a multivariate regression model. *Results*. Between 12/1988 and 8/2005, 1444 patients were enrolled and data current to 2001, 2004, or 2008 were used. Patients were with a median age of 12 years (0–45), 55% male and 88% Caucasian. The 5-year EFS was 68.3% ± 1.3%. In univariate analysis age, treatment, and tumor location were identified for inclusion in the multivariate model, and all remained significant (*p* < 0.01). Since tumor size was not collected in the last study, the other two were reanalyzed. This model identified age, treatment, tumor location, and tumor size as significant predictors. *Conclusion*. Age > 18 years, pelvic tumor, size > 8 cms, and chemotherapy without ifosfamide/etoposide significantly predict worse outcome. AEWS0031 is NCT00006734, INT0091 and INT0054 designed before 1993 (unregistered).

## 1. Introduction

The 5-year event-free survival (EFS) for nonmetastatic Ewing sarcoma patients has improved to 60–70% [1–6], but 2-year survival for patients that relapse is 20–30% [7, 8]. Identifying factors predicting relapse may help develop new strategies for those patients. Other studies have consistently identified tumor location [3, 9–12] and age [9, 11–15] as EFS predictors. Factors less consistently identified include lactic dehydrogenase [9, 10, 13], histologic response [9, 13, 14, 16], tumor volume [3, 6, 13, 14, 16, 17], tumor size [12, 18], and surgical margins [19]. Histological response and surgical margin accurately predict risk of recurrence but because they can only be determined after chemotherapy and surgical resection, they cannot help identify potential new strategies for patients at the time of diagnosis.

The Children's Oncology Group (COG) and its predecessors conducted three studies (1988–2005) for newly diagnosed localized Ewing sarcoma (ES) or peripheral primitive neuroectodermal tumor (PNET) of bone [1, 2, 5] and/or soft tissue. These studies included 1444 patients and used common chemotherapy agents. Most of the protocols' eligibility criteria overlapped and the schedule and evaluation methods were similar. They carried forward “standard” therapies for comparison. This series represents a group of patients who are homogeneous except for the patient/treatment factors examined in this analysis, reducing the likelihood that observed associations are attributable to historical changes in patients or evaluation methods.

We sought to pool the high-quality dataset represented by those COG trials [1, 2, 5] to identify (1) demographic (i.e., age and sex), (2) treatment-related (assigned chemotherapy), and (3) tumor-related factors (tumor size and location) associated with EFS.

## 2. Patients and Methods

### 2.1. Studies

**Figure 1:**
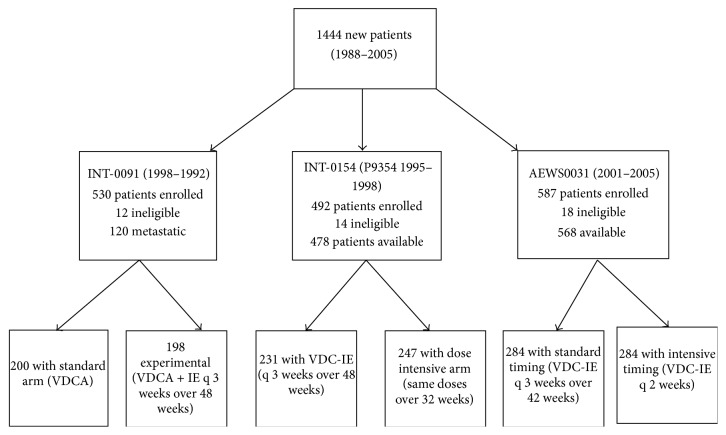
CONSORT diagram for 1444 patients enrolled in three consecutive COG studies. The arms to which patients were randomized are shown along with exclusion criteria and those patients who were thereby ineligible. CONSORT = Consolidated Standards of Reporting Trials; COG = Children's Oncology Group.

We analysed selected factors at study enrolment for 1444 patients treated in three COG studies: (1) INT-0091 [1]; (2) INT-0154 [2]; and (3) AEWS0031 [5] (Figure 1). The INT-0091 study enrolled patients between 1988 and 1992 [1]; the INT-0154 enrolled patients between 1995 and 1998 [2]; while the most recent study (AEWS0031) enrolled patients between 2001 and 2005 [5]. Details of the three studies have been previously published [1, 2, 5].

Briefly, patients < 30 years of age with newly diagnosed, histologically confirmed primary ES or PNET were eligible for enrolment in INT-0091 [1]. Eligible patients were randomized to treatment with vincristine, doxorubicin, and cyclophosphamide (VDC), with or without ifosfamide and etoposide (IE). Although the study included patients with metastatic disease, those patients are not considered in our analysis. Patients < 30 years with newly diagnosed, histologically confirmed ES or peripheral PNET of bone or soft tissue were eligible for enrolment in INT-0154 and were randomized to dose-intensified or standard therapy (Figure 1) [2]. The dose-intensified arm of the study administered the same total doses of chemotherapy every 3 weeks over 32 rather than 48 weeks. Patients < 50 years with newly diagnosed, histologically confirmed ES or peripheral PNET of bone or soft tissue were eligible for enrollment in AEWS0031 and were randomized to receive the same chemotherapy doses every 2 weeks (dose-dense) or every 3 weeks (standard therapy) [5].

### 2.2. Treatment

#### 2.2.1. Chemotherapy

The standard chemotherapy doses included the following: VCD—vincristine 2 mg/m^2^ (2 mg maximum dose), doxorubicin 75 mg/m^2^/dose (either as a single bolus, two daily boluses, or 48 h continuous infusion), and cyclophosphamide 1200 mg/m^2^, followed by mesna uroprotection. Dactinomycin 1.25 mg/m^2^/d was substituted for doxorubicin when a total doxorubicin dose of 375 mg/m^2^ was reached in INT-0091. Courses of IE included ifosfamide 1800 mg/m^2^/d for 5 days, given with mesna uroprotection and etoposide 100 mg/m^2^/d over the same 5 days.

#### 2.2.2. Local Control

The studies prescribed local control following 12 weeks of therapy, which in the first two studies [1, 2] and in the control arm of the third study included four cycles of chemotherapy but followed 6 cycles in the experimental arm (every-2-week treatment) of the third study [5]. Although the choice of local control was left up to the treating physician, all protocols provided guidelines [1, 2, 5]. The protocols allowed surgery for tumors deemed resectable. For radiotherapy alone, the initial tumor volume (soft-tissue and osseous tumor extent) with a 3 cm margin was treated with 4500 cGy, followed by reduction in treatment volume to the postchemotherapy, preradiotherapy tumor for 1080 cGy more (total dose 5580 cGy). A smaller margin was allowed to avoid radiation to the epiphysis. Patients with residual tumor after surgery were irradiated using the dose-volume guidelines for gross residual disease; for microscopic residual disease, irradiation was limited to 4500 cGy to the original volume with a 1 cm margin. No supplemental radiotherapy was administered to patients achieving a complete resection of the primary tumor with clear margins regardless of extent of necrosis or tumor size. For patients with extraosseous tumors and a complete response to induction chemotherapy, the initial tumor volume plus a 2 cm margin received 4500 cGy followed by a boost of 540 cGy with a 1 cm margin (total dose 5040 cGy).

### 2.3. Statistical Methods

We defined EFS as the time from study entry until the occurrence of an analytic event or date of last contact, whichever came first. An analytic event was defined as disease progression, diagnosis of second malignant neoplasm (SMN), or patient death prior to the development of disease progression or SMN. A patient who had not experienced an event by the date of last follow-up was censored.

Exploratory analysis was complicated by the fact that some data were not collected in all studies and even when intended to be collected some data were missing. The major sources and types of missing data were as follows: (1) INT-0091 excluded patients with extraosseous ES from enrollment; (2) in AEWS0031 tumor size was not collected; and (3) tumor size was not reported for 266 participants in the other two studies. We therefore excluded tumor size and soft-tissue ES from our first multivariate model. Participants whose data were truly missing (request for data present but not provided by the institutional investigators) were also eliminated from the analysis.

The distributions of EFS and overall survival were estimated by the method of Kaplan and Meier [20]. Risk of adverse event was compared across groups, defined by treatment or prognostic factors using the log-rank test. Comparisons involving the chemotherapy randomizations were conducted with patients' outcomes assigned to their randomized treatment arm at enrollment (intent-to-treat analyses). The prognostic significance and associated RR for various patient characteristics measured at study entry were assessed by a proportional hazards regression model with the characteristic of interest as the only component. Confidence intervals (CI) for RRs were derived from the proportional hazards regression model [21]. In addition, a likelihood ratio test was performed to confirm the homogeneity of model parameters across studies. The likelihood ratio test statistic was constructed by comparing the likelihood from the stratified Cox regression model fitting common risk coefficients stratified by study (assumes model parameters are not different across strata) with the likelihood of the Cox regression fitting study-specific risk coefficients.

Only potential prognostic factors measured at study enrollment were assessed in the dataset. In particular, the relationship between risks of an event and death and local control modalities were not analyzed. The potential prognostic factors considered included the following: (1) patient age at enrollment; (2) site of primary tumor; and (3) patient sex. Age at enrollment was categorized.

All the factors noted above were explored further to assess their relative prognostic effects when considered jointly. To determine whether tumor size had an impact on outcome using COG-directed therapy, we performed a second multivariate analysis including only patients enrolled in INT-0091 and INT-0154, where tumor size in two perpendicular dimensions was requested but not required for eligibility. This allowed us to determine whether tumor size was an independent predictor of EFS using US treatment (in COG). For this analysis, tumor size was categorized.

## 3. Results

A total of 1444 patients were enrolled in INT-0091, INT-0154, and AEWS0031 between December 1988 and August 2005 (Figure 1). The INT-0091 trial opened in December 1988 and closed in November 1992. Data current to August 2001 were used for our analysis, which included 395 eligible patients. The INT-0154 trial opened in March 1995 and closed in September 1998. Data current to December 2006 were used for our analysis, which included 477 eligible patients. The AEWS0031 trial opened in May 2001 and closed in August 2005. Data current to March 2009 were used for our analysis, which included 568 eligible patients. Baseline characteristics included a median age of 12 years (range, 0–45), and 55% of patients were male (see Table 1 for details).

**Figure 2:**
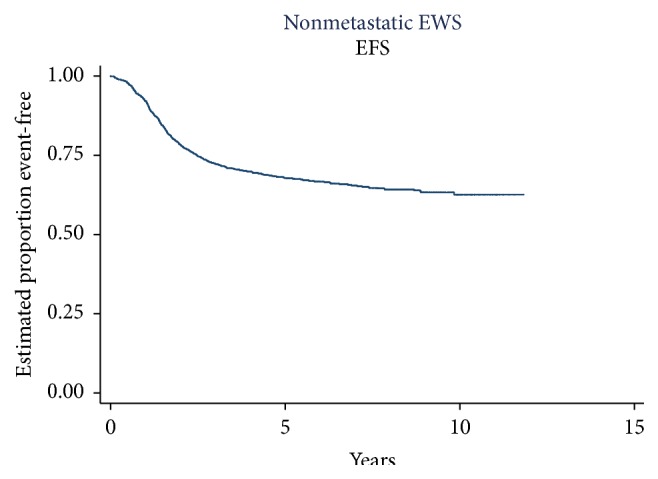
EFS for 1444 patients enrolled in consecutive COG trials. The 5-year EFS for the group was 68.32%  ± 1.3%. EFS = event-free survival; COG = Children's Oncology Group.

The 5-year EFS for the 1444 patients enrolled in the three studies was 68.3%  ± 1.3% (Figure 2). The 5-year EFS for INT-0091, INT-0054, and AEWS0031 was 61.5%  ± 2.5%, 71%  ±  2.1%, and 70%  ± 2.6%, respectively. The risk for an EFS event differed significantly across studies (*p* = 0.0071). This is likely driven by the absence of IE in the control arm of INT-0091, since INT-0154 and AEWS0031 have very similar EFS.

As seen in Table 2, the RR of an event using the intensively timed treatment of AEWS0031 relative to the standard timing is 0.75 (0.5–1.01) confirming that intensively timed therapy reduced event risk compared to standard-dose-and-timing IE or non-IE containing treatment across the studies. In this pooled analysis, the confidence intervals cross 1 (*p* = 0.061) and are just outside conventional statistical significance. Additionally, the RR of an event using the non-IE arm of INT-0091 relative to the standard timing arm of AEWS0031 is 1.5 (1.12–2). This confirms the inferiority of non-IE containing regimens as the risk of an event is 50% higher in those patients. The RRs are similar for patients in the standard timing IE treatments.

Univariate analysis identified three variables for consideration in assessing the relative values of prognostic factors measured at study enrollment (Table 3): (1) patient age at enrollment (≤9 years, 10–17 years, and ≥18 years); (2) assigned treatment (intensive-timing IE, standard timing IE, and non-IE); and (3) tumor location (pelvis, nonpelvic bone).

Table 4 presents the results of the multivariate analysis including the estimated risk coefficients and 95% confidence intervals. Patient age at enrollment remains a significant predictor of EFS, and patients ≥ 18 years have greater than a twofold increased risk of an event (RR 2.14 (CI 1.59–2.87, *p* = 0.000)) compared to patients ≤ 9 years.

Tumor location and assigned treatment also retain their role as significant predictors of EFS in the presence of one another. Patients with a pelvic tumor have a higher event risk RR 1.34 (1.07–1.67) than patients with nonpelvic tumors. Assigned treatment was also an important predictor of outcome and patients treated with non-IE containing treatment had an increased event risk RR 1.84 (1.33–2.53). In our multivariate analysis, risk of event was unrelated to patient sex. The estimates of the effects of age, tumor site, and treatment did not differ significantly between trials (*p* = 0.2587).

We also evaluated whether tumor size and tumor location were both predictive of outcome by performing a second multivariate analysis including only patients treated in INT-0091 and INT-0154. As shown in Table 5, age, tumor location, non-IE treatment, and tumor size were all significant predictors of EFS. In this analysis patients ≥ 18 years have a twofold event risk compared to younger patients (RR 1.97 (1.33–2.93)). Patients treated with a non-IE regimen have a 56% higher event risk than those treated with IE regimens (RR 1.56 (1.19–2.05)). Importantly, both pelvic tumor location and tumor size are predictors of EFS in the presence of each other. Patients with pelvic tumors have a 44% increased risk of an event RR 1.44 (1.07–1.92) while patients with tumors > 13 cm have an event risk twofold higher than patients with tumors < 8 cm RR 2.00 (1.43–2.79).

## 4. Discussion

Previous studies have identified tumor location [3, 9–12] and age [9, 11–15] as consistent predictors of poor EFS. The two large series (>500 patients) [11, 13] assessing factors predicting relapse were both based on the European treatment approaches. The chemotherapy treatment and local control approaches in Europe differ from those in the United States. For instance, European investigators stratify patients based on tumor size [16, 17] and consider histological response to be the most important predictor of outcome [16, 22]. Therefore, we thought it important to evaluate demographic, treatment, and tumor characteristics for their impact on EFS using a large dataset of US treated patients.

Our study had several limitations related to its retrospective nature, including the fact that some patient characteristics were not missing at random. These included tumor size (not collected in AEWS0031) and patients with soft-tissue tumors (not included in INT-0091). We excluded these factors from our first multivariate model rather than employ methods to impute the missing information and adjust *p* values and confidence intervals accordingly. We also excluded any participants with missing data from our analysis. Our second multivariate analysis included only patients in INT-0091 and INT-0154 since tumor size was part of the data collection. This reduced dataset represents individuals where the initial data collection strategy included assessment of maximum tumor dimension. We consider this approach optimal for evaluating the relative importance of tumor size and tumor location in our studies.

We were able to confirm the importance of age ≥ 18 years as an independent predictor of worse EFS. Though older age has been consistently identified as predicting higher event rates in multiple series [9, 11, 13, 14], the age cutoff has varied from 12 to 15 years among those studies. This variability likely reflects the eligible patient population included in these studies. Additionally, a small study reported that older patients did as well as younger patients if treated with similar therapy [23]. Our analysis indicates that in the context of US-style therapy age ≥ 18 years is an important predictor of worse EFS. Our conclusions are limited by the small number of patients older than 25 years; therefore, continued enrollment of patients ≥ 18 years in randomized controlled trials will help further characterize the optimal age cutoff for predicting EFS.

Tumor location also predicted increased event risk; patients with pelvic primaries were particularly at high risk. This finding agrees with other studies of ES [1, 2, 11, 12, 15, 24] and is consistently used as a stratification factor in both European and North American studies. Determining whether the treatment of patients with pelvic tumors is better if they are treated with the combination of surgery and radiotherapy is difficult since randomization for this subset would not be feasible. European studies more frequently use a combination of surgery and radiotherapy for such patients. This strategy is one not frequently used in the United States, and EFS is similar suggesting that both approaches may be equivalent.

Assigned chemotherapy treatment was an important predictor of EFS in our study. As expected from results of our previous trials [1, 2], the EFS for patients treated with high- and standard-dose IE did not differ, but patients receiving non-IE containing therapy had a higher risk of an event with RR 1.63 (1.29–2.06). The use of IE with VDC has become part of the US standard treatment for patients with ES [1, 2, 5]. Additionally, in our analysis the RR of an event for patients treated with dose-dense therapy was 0.89 (0.67–1.16), consistent with a protective effect. However, this result does not achieve conventional statistical significance. The reference group (standard timing IE) is heterogeneous in that it included patients treated with increased doses of ifosfamide and cyclophosphamide. This is in contrast to the results of the COG randomized trial, which confirmed the importance of dose-dense therapy in improving EFS [5]. This phenomenon has been well documented in the literature and supports the contention that randomized controlled trials are the preferred method to evaluate the prognostic significance of interventions, where control can be exercised over factors that could confound the statistical comparison [25].

We were able to document the importance of tumor size in the context of tumor location in our second multivariate analysis (limited to INT-0091 and INT-0154), which revealed a twofold increased risk of an event for patients with tumors larger than 13 cm (RR 2.00 (1.44–2.79)). On average, patient risk appeared to increase with larger tumor size. The size of our population limited our sensitivity to these more subtle differences. Tumor size and/or volume have been identified in a number of studies as significant predictor of event risk [1, 3, 11–13, 16, 17] and have been incorporated into the current Euro-Ewing protocol where patients with estimated tumor volume greater than 200 mL [16, 17] are considered at high risk and are eligible for randomization to continuing standard chemotherapy versus intensification with the use of stem-cell transplant. The tumor cutoff of 200 mL used in European studies [13, 16, 17] is a smaller volume than the greater than 8 cm size used in North America (which corresponds to a 268 mL spherical tumor) [1, 12].

In conclusion, this is the largest series of US treated ES patients receiving a similar therapy backbone. We confirmed that primary tumor site (in the pelvis) and age ≥ 18 years are predictive of an increased event risk and should be considered at the time of treatment assignment. Patients ≥ 18 years and those with pelvic tumors might benefit from new treatment strategies. For example, chemoradiotherapy may allow resection for a larger number of pelvic tumors. Larger tumor size is also a predictor of worse EFS and should be included as a stratification factor in future US trials.

## Figures and Tables

**Table 1:** Characteristics of 1444 patients enrolled in COG studies.

Factor	Study			
	INT-0091	INT-0154 (P9354)	AEWS0031	Total
Number of patients	398	478	568	1444
Median age, years (range)	12 (0–28)	12 (0–30)	12 (0–45)	12 (0–45)
<9 years, *n* (%)	121 (30)	148 (31)	162 (28)	431 (29.9)
9–18 years	227 (57)	265 (55)	339 (60)	831 (57.6)
>18 years	50 (13)	65 (14)	67 (12)	182 (12.6)
Sex, %				
Men	57	55	54	55
Women	43	45	46	45
Primary sites, *n* (%)				
Appendicular	188 (47)	175 (36)	195 (34)	558 (39)
Thoracic	69 (17)	75 (16)	89 (16)	233 (16)
Pelvic	93 (23)	70 (15)	90 (16)	253 (18)
Other axial	48 (12)	57 (12)	75 (13)	180 (12)
Extraosseous	—	94 (20)	119 (21)	213 (15)
Missing	0 (0)	7 (1.5)	0 (0)	7 (0.48)
Tumor size, *n* (%)				
<8 cm	155 (30)	141 (29.5)	0 (0)	296 (10)
9–12 cm	113 (28)	98 (21)	0 (0)	211 (15)
>13 cm	64 (16)	39 (8.2)	0 (0)	93 (7)
Not reported	66 (17)	200 (42)	568 (100)	834 (57.8)

COG = Children's Oncology Group.

**Table 2:** EFS risk by treatment arm relative to standard treatment in AEWS0031.

Treatment	Hazard ratio	Std. error	*Z*	*p* > *z*	Confidence intervals	
AEWS0031-ST	1.00					
AEWS0031-IT	0.7488	0.1157	−1.87	0.061	0.5532	1.0135
INT-0154-HD	0.8805	0.1339	−0.84	0.4020	0.6536	1.1861
INT-0154-SD	0.8034	0.1271	−1.38	0.1660	0.5891	1.0955
INT-0091-IE	0.9613	0.1527	−0.25	0.8040	0.7042	1.3123
INT-0091-Std.	1.5064	0.2212	2.79	0.0050	1.1296	2.0088

**Table 3:** Estimated risk coefficients on univariate analysis for 1444 patients treated in consecutive COG studies.

Factor	Characteristic	Relative risk	95% confidence interval	*p* value compared reference	Global *p* value
Age yrs. (ref. <9)					0.0000
	10–17	1.34	1.08–1.66	0.0090	
	18+	2.35	1.78–3.11	0.0000	
Primary site (ref. pelvis)					0.0002
	Nonpelvic tumor	0.70	0.52–0.93	0.0140	
	Extraosseous	0.52	0.37–0.72	0.0000	
Gender (ref. male)					0.3404
	Female	0.92	0.77–1.10	0.08	
Treatment (ref. standard timing)					0.0002
	Non-I/E	1.65	1.31–2.09	0.0000	
	Intensive timing	0.82	0.64–1.06	0.1260	
Tumor size, cm (ref. <8)					0.0002
	9–12 cm	1.26	0.96–1.65	0.0990	
	>13 cm	1.98	1.45–2.70	0.0000	

**Table 4:** Estimated risk coefficients for multivariate analysis excluding extraosseous patients (*N* = 1231).

Factor	Characteristic	Relative risk	95% confidence interval	*p* value compared to reference	Global *p* value
Age yrs. (ref. ≤9)					0.0000
	10–17	1.24	0.98–1.55	0.070	
	18+	2.14	1.59–2.87	0.000	
Primary site (ref. nonpelvic)					0.0110
	Pelvic tumor	1.34	1.07–1.67	0.009	
Treatment (ref. intensive timing)					0.0001
	Standard timing	1.13	0.86–1.48	0.384	
	Non-I/E	1.84	1.33–2.53	0.000	

**Table 5:** Estimated risk coefficients for patients treated in INT-0091 and INT-0154 (P9354) [excluding patients in AEWS0031] (*N* = 716).

Factor	Characteristic	Relative risk	95% confidence interval	*p* value compared reference	Global *p* value
Age yrs. (ref. ≤9)					0.0043
	10–17	1.27	0.93–1.72	0.130	
	18+	1.97	1.33–2.93	0.001	
Primary site (ref. nonpelvic)					0.0173
	Pelvic tumor	1.44	1.07–1.92	0.014	
Treatment (ref. standard timing)					0.0019
	Non-I/E	1.56	1.19–2.05	0.001	
Tumor size, cms (ref. <8)					0.0005
	9–12 cm	1.24	0.93–1.65	0.137	
	>13 cm	2.00	1.43–2.79	0.000	
